# Two Women Presenting Worsening Cutaneous Ulcers during Pregnancy: Diagnosis, Immune Response, and Follow-up

**DOI:** 10.1371/journal.pntd.0002472

**Published:** 2013-12-12

**Authors:** Fátima Conceição-Silva, Fernanda Nazaré Morgado, Maria Inês Fernandes Pimentel, Erica de Camargo Ferreira e Vasconcellos, Armando O. Schubach, Cláudia M. Valete-Rosalino, Pascale Kropf, Ingrid Müller

**Affiliations:** 1 Laboratório de Imunoparasitologia, Instituto Oswaldo Cruz, Fiocruz, Brasil; 2 Laboratório de Vigilância em Leishmanioses, Instituto de Pesquisa Clinica Evandro Chagas, Fiocruz, Brasil; 3 Departamento de Otorrino e Oftalmologia, Faculdade de Medicina, Universidade Federal do Rio de Janeiro, Rio de Janeiro, Brasil; 4 Imperial College London, Faculty of Medicine, Section of Immunology, London, United Kingdom; 5 Imperial College London, Faculty of Medicine, Section of Immunology, London, United Kingdom; University of Notre Dame, United States of America

## Description of Cases

Two pregnant patients (PP) presenting cutaneous ulcers were referred to our outpatient clinics at Instituto de Pesquisas Clínicas Evandro Chagas (IPEC)–Fiocruz. The first patient (PP1, 24 years old) had an ulcer above the knee (35×25 mm). The second patient (PP2, 25 years old) presented an ulcer (22×17 mm) on the left arm. Lesion onset occurred approximately 2 months earlier in both patients, and both were 2–3 months pregnant. As they lived in areas endemic for American cutaneous leishmaniasis (ATL) and sporotrichosis (SP), specific tests for ATL and SP (lesion biopsies: histopathology, immunohistochemistry [IHC], and parasite/fungi/bacteria isolation; peripheral blood: serology) were performed. Routine pregnancy screenings were done, and blood counts and biochemistry were within the normal range.

## Diagnosis Confirmation and Follow-up

**Figure 1:**
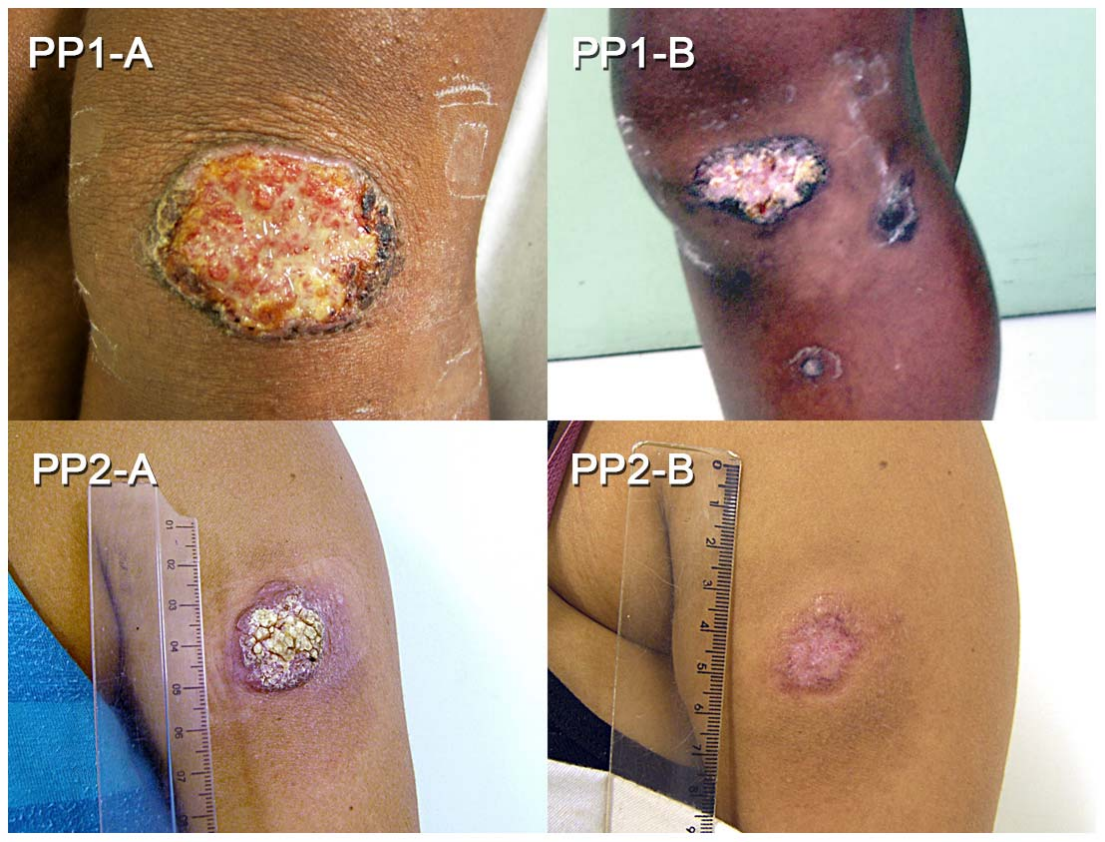
Cutaneous lesions in ATL patients during pregnancy and post delivery. The upper left picture shows the lesion of pregnant patient 1 (PP1-A) at 8 months of pregnancy, and the upper right shows the lesion of the same patient 3 months after birth (PP1-B). The lower left picture shows the lesion of pregnant patient 2 (PP2-A) at 6 months of pregnancy, and the lower right picture shows the lesion of this ATL patient 6 months post delivery (PP2-B). Lesions during pregnancy developed as ulcerative-vegetative (A), and post delivery, signs of lesion healing (epithelialisation, hyperpigmentation, and fibrosis) are seen (B).

**Figure 2:**
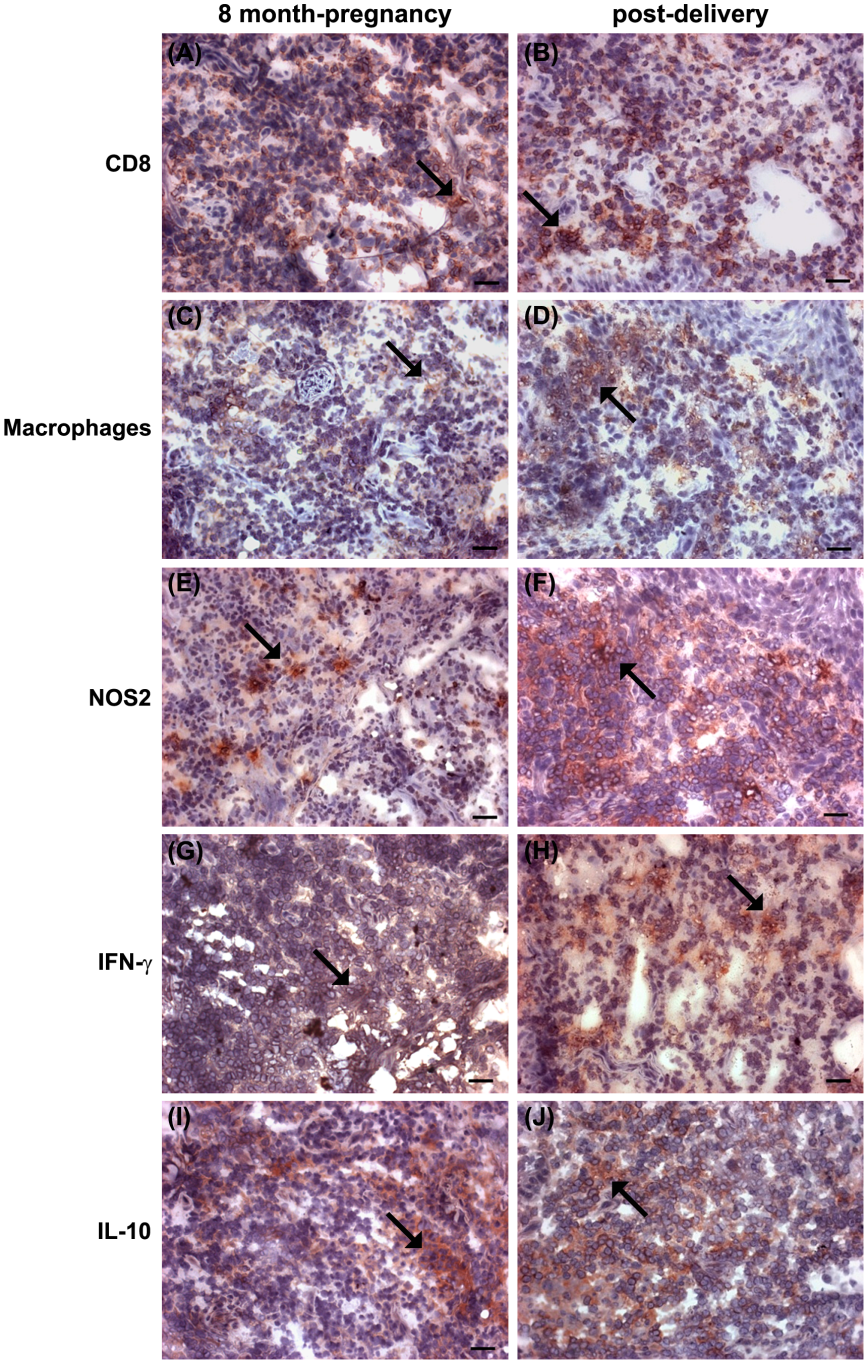
Inflammatory reaction in active cutaneous lesions of untreated ATL patient (PP1) during pregnancy and postpartum. The inflammatory response in the lesions was assessed by in situ immunohistochemistry. (A,B) CD8^+^ cells, (C,D) CD68^+^ cells, (E,F) NOS2, (G,H) IFN-γ, and (I,J) IL-10 expression in active lesions of pregnant ATL patient (PP1). (A,C,E,G,I) at 8 months of pregnancy; (B,D,F,H,J) at 2–6 months post delivery; Magnification: 200× (bar = 50 µm); arrows indicate examples of positive areas.

The diagnosis for both patients was localized ATL (LCL), since *Leishmania* parasites, characterized as *Leishmania braziliensis*, were isolated from lesions, whereas isolation of fungi and bacteria (including mycobacteria) was negative. This was confirmed by histopathology and IHC. As the patients were pregnant, specific anti-leishmanial treatment was not started, and the patients were monitored monthly. The lesion of PP1 increased in size (50×37 mm) and became ulcerative-vegetative at 5 months of pregnancy, and towards the end of pregnancy (8–9 months), small satellite ulcers appeared (not shown) around the main lesion (54×40 mm) (Figure 1 PP1-A). No other skin or mucosal lesions were detected. About 2 months after delivery, the exuberance of the lesions had decreased; they became shallower and less secreting. At 3 months postpartum, satellite lesions healed, and the beginning of peripheral epithelialisation with hyper-pigmentation areas was observed (Figure 1 PP1-B). This clinical evidence of spontaneous improvement after delivery was confirmed by IHC (Figure 2). An inflammatory granulomatous infiltrate with areas of fibrosis and epithelialisation was detected. Since the patient decided to continue breastfeeding, treatment with pentavalent antimony was not started. Clinical cure occurred 3 months later, and the lesions remained healed 48 months later without any sign of mucosal metastasis.

The lesion of PP2 also developed as ulcerative-vegetative and increased in size until 6 months of pregnancy (40×26 mm), when the lesion size stabilized (Figure 1 PP2-A). At 2 months postpartum, the area measured 27×20 mm; it was epithelialised and completely healed 6 months postpartum (Figure 1 P2-B). At this time, histopathology showed the presence of fibrous tissue with focal residual inflammatory cells, and no parasites were detected. The patient did not receive specific treatment, and 12 months after delivery, the area presented an atrophic scar measuring 26×25 mm. No signs of mucosal lesions were detected, and *Leishmania* serology was negative.

## Why Do ATL Lesions Tend to Worsen during Pregnancy?

The changes in innate and adaptive maternal immune responses during pregnancy are tightly linked to gestation and normalize postpartum [1]. These transient changes can have both beneficial and detrimental consequences, as exemplified by improved rheumatoid arthritis and systemic lupus erythematosus [2] and enhanced susceptibility to infection or aggravated disease during pregnancy [3]. However, understanding of the impact of parasitic diseases on mother and infant is still limited.

Some aspects of the maternal immune response to *Leishmania* spp during pregnancy have been studied in animal models and showed that *Leishmania*-infected pregnant C57BL/6 mice developed larger lesions, harbored increased parasite burdens, and expressed less IFN-γ but increased levels of IL-4, IL-5, and IL-10 as compared to nonpregnant controls [4], [5]. In this model, IFN-γ and TNF-α production by placental cells correlated with fetal resorption and implantation failure [6]. These data suggest that anti-leishmanial Th1 responses could adversely affect the success of pregnancies, either by interfering with placental development and maintenance and/or by impairing preimplantation events. These results imply that the modulation of maternal immune responses during pregnancy favors parasite growth and lesion development. Furthermore, control of *Leishmania panamensis* infection during pregnancy in a hamster model was mostly due to innate immune responses, including enhanced estrogen-mediated up-regulation of NOS2 expression and NO production [7].

Human pregnancy has been associated with a shift in Th1/Th2 balance, and pregnant woman exhibit reduced type 1 responsiveness, which is considered to be important for healing in leishmaniasis. Indeed, atypical manifestations and worsening of LCL during pregnancy is consistent with this interpretation, but the mechanism is not fully characterized.

## Can We Detect Changes in Immune Responses of Pregnant ATL Patients?

To answer this question, we tested local (lesion) and systemic (blood) immune responses. Punch biopsies from cutaneous lesion (5–6 mm) and/or peripheral blood samples (EDTA) were collected in the second, fifth, and eighth month of pregnancy and 2–6 months after delivery. We also tested tissue and blood from six nonpregnant, age-matched ATL patients before treatment (Table S1), as well as peripheral blood of ten healthy, age-matched female volunteers (25.5±0.93 years).

This study was approved by the Ethics Committee in Human Research (CEP–IPEC–Fiocruz–number 014/2001). All participants gave written informed consent.

**Figure 3:**
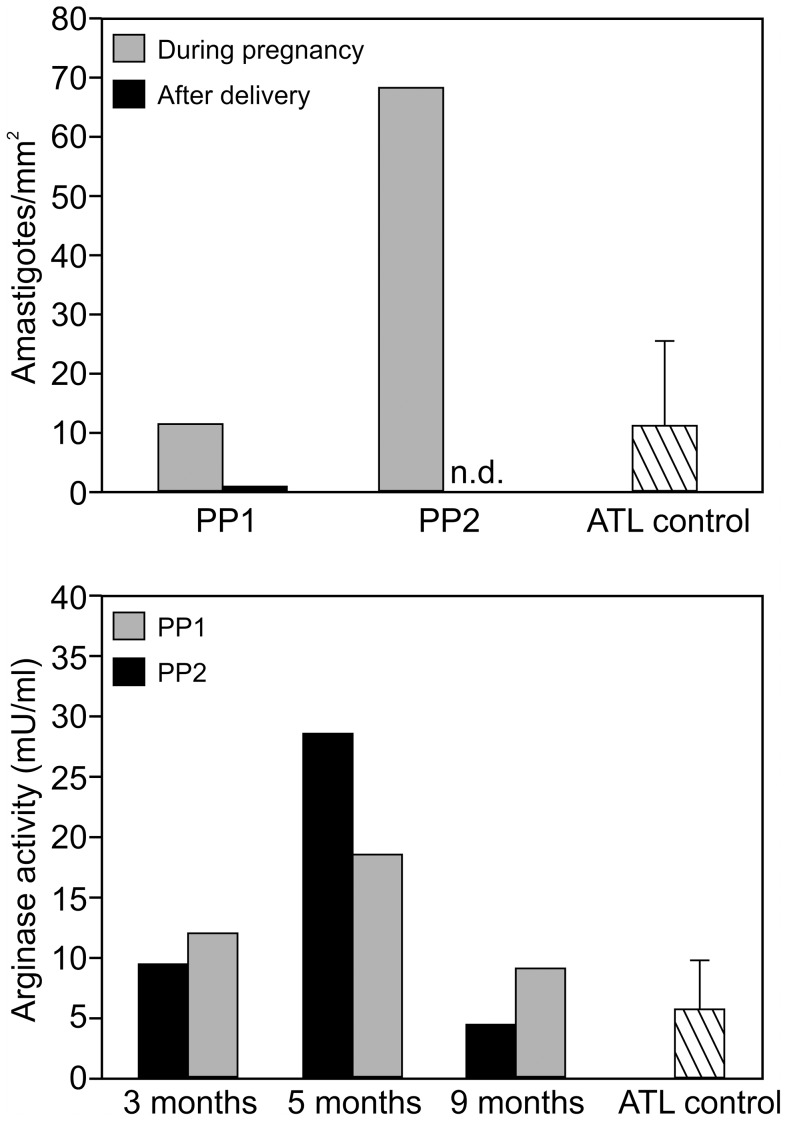
(Upper panel) Parasite enumeration during pregnancy and postpartum. Amastigotes present in the lesions were enumerated by in situ immunohistochemistry in active cutaneous lesions of both pregnant patients (PP1, PP2) before treatment, in the eighth month of pregnancy (gray bars) and 2–6 months after birth (black bars). Amastigote numbers present in lesions of nonpregnant ATL patients (average of three patients) before treatment are shown as comparison. The results are expressed as number of parasites per mm^2^, using a grid-scale consisting of 20×20 subdivisions in an area of 10 mm^2^×10 mm^2^, adapted to slides as previously described [24]. The results shown are median ± SEM. n.d. = not detectable; (Lower panel) Serum arginase activity. Arginase activity (U/mL) was determined in sera obtained from peripheral blood of pregnant ATL patients collected at 3, 5, and 9 months of pregnancy (black bar PP1; grey bar PP2) as well as four age-matched nonpregnant ATL patients (hatched bars). The results shown are median ± SEM.

The in situ inflammation was evaluated by IHC as described [8] at the eighth month of pregnancy and at 2–6 months postpartum as well as in the nonpregnant ATL group before treatment (Figure 2, Table S2). Despite similarities in organization and architecture of the inflammatory site during pregnancy and after birth, the concentration and distribution of some cell types and secretory products changed in both pregnant patients after delivery. T lymphocytes and macrophages were abundant at both times (Figure 2A–2D). Postpartum, a pronounced increase in both extent and number of NOS2 positive areas was detected (Figure 2E–2F). In accordance with increased NOS2, parasites were no longer detectable in the lesion postpartum (Figure 3 upper panel). Although the distribution of IL-10 positive areas remained similar on two occasions (Figure 2I–2J), there was an increase of IFN-γ positive areas postpartum (Figure 2G–2H). At the same time, an increase in the percentage of B cells, neutrophils, Bax^+^ cells, CD25^+^, and Foxp3^+^ cells was observed. However, Bcl-2^+^ and Ki-67^+^ cells were reduced. In the eighth month of pregnancy, the patients expressed less IFN-γ than nonpregnant female ATL patients (Table S2).

The enzymatic activity of arginase present in serum was measured as previously described [9]. The enzyme arginase, chiefly expressed in human neutrophils and macrophages, catabolizes its substrate L-arginine, and under certain conditions, increased arginase activity causes reduction in L-arginine levels in the extracellular fluid. Reduced availability of L-arginine can impair T cell responses and weaken immune responses against infection [10]. We previously reported enhanced arginase activity in pregnant women and wished to test the hypothesis that worsening of leishmaniasis in pregnancy is associated with increased arginase activity [9]. Results presented in Figure 3 (lower panel) show that in both pregnant patients, arginase activity increased during pregnancy and declined at time of birth to levels similar to those observed in ATL nonpregnant controls.

**Figure 4:**
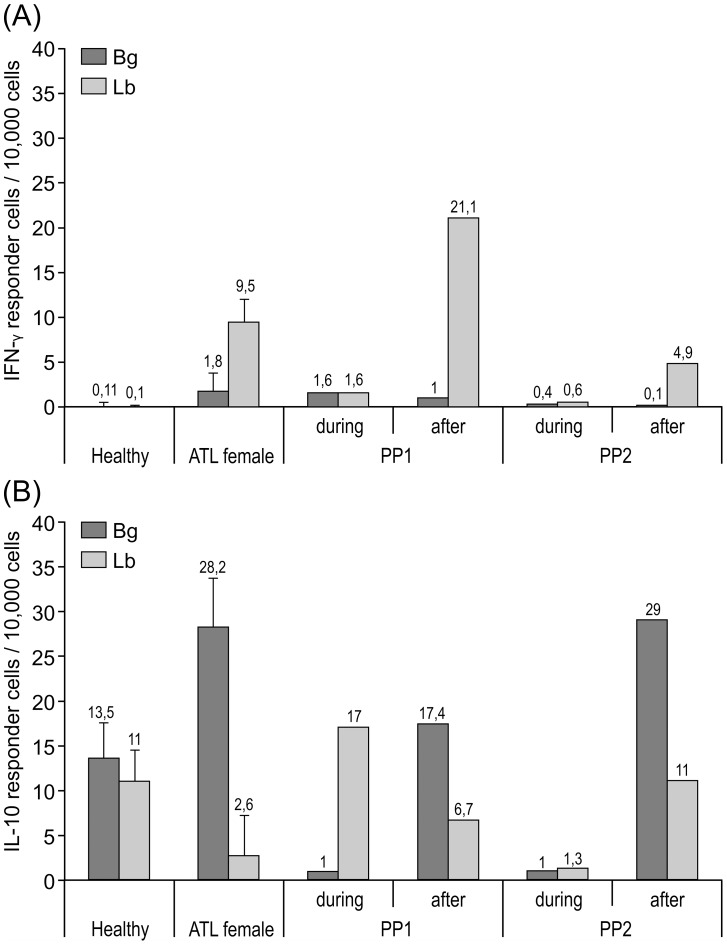
Determination of IFN-γ and IL-10–producing cells in the peripheral blood during pregnancy and postpartum. (A) The number of IFN-γ and (B) of IL-10–producing cells in peripheral blood was determined at 8 months of pregnancy and 2–6 months after birth in both pregnant ATL patients by ELISPOT assay and compared to those present in nonpregnant ATL patients before specific treatment and in healthy volunteers. Spontaneous release (black bars) as well as antigen-specific responses to restimulation with *Leishmania*-antigen in vitro (grey bars) are shown. The results from antigen-stimulated PBMC were expressed as the difference of the number of spots/10,000 PBMC in the antigen-stimulated wells minus the mean number of spots in control wells (with medium alone = spontaneous release) from the same donor. The results shown are median ± SEM.

Antigen-specific cytokine secretion by PBMC was assessed by ELISPOT assay at 8 months of pregnancy and 2–6 months after birth, as well as in nonpregnant ATL patients before treatment and in healthy volunteers. IFN-γ secretion was decreased in both patients during pregnancy but increased postpartum, reaching values comparable to those seen in untreated nonpregnant ATL patients (Figure 4A). Furthermore, high frequencies of IL-10–producing cells were detected in the absence of antigen that decreased following antigenic restimulation in ATL patients and in ATL pregnant patients after birth. However, antigen-specific IL-10–producing cells increased in response to specific restimulation during pregnancy (Figure 4B). Thus, the results obtained from lesion tissues and peripheral blood show changes in both local and systemic maternal immune responses. After delivery, the healing of lesions correlated with increased in situ NOS2 expression (Figure 2F), decreased parasite counts (Figure 3 upper panel), as well as increased systemic and in situ expression of IFN-γ (Figure 4A).

## Should ATL Be Treated during Pregnancy?

Studies on different maternal infections have shown that mother and offspring can have distinct susceptibilities to infection in each stage of reproduction, from conception and gestation to parturition and the neonatal period. With regard to infections with protozoans, maternal malaria has been associated with fetal infection, preterm delivery, and growth restriction as well as maternal per partum death due to hemorrhagic complications [3]. The impact of the time of gestation on the consequences of infection was shown in toxoplasmosis: severe congenital toxoplasmosis was associated with maternal infection starting in the second trimester of pregnancy, and infections in the first trimester were associated with fetal loss due to severe damage to the embryo [11]. To our knowledge, there are no publications addressing these issues in cutaneous leishmaniasis (CL) patients. Untreated visceral leishmaniasis (VL) during pregnancy puts maternal life at risk and may result in fetal loss or in congenital VL [12], [13], and therefore, treatment of VL during pregnancy is compulsory. However, treatment of pregnant ATL patients is a debated issue [12], [14], [15] since there is no description of congenital infection, and many antileishmanial drugs, such as pentavalent antimony or miltefosine, are teratogenic [15]–[18]. Consequently, alternative therapies should be evaluated in order to warrant safety and efficacy in this group of patients. Guimarães et al [19] showed that 40% of patients with atypical manifestation of ATL were pregnant women and suggested that Amphotericin B should be considered as the drug of choice for all patients diagnosed with atypical ATL. Intralesional treatment with meglumine antimoniate was successful in over 83% of patients treated who had contraindication to systemic therapy [20], but there is a lack of evidence demonstrating safety in pregnant women. As spontaneous healing has been reported to occur after delivery [21], several groups avoid the use of specific treatment and follow the patients by using local heating and/or antibiotic ointments to control lesion development and secondary infections. More work is required to optimize the management of ATL during pregnancy.

## Is the Occurrence of Metastases Possible?

The mechanism of metastasis in ATL is not fully understood. In Brazil, 3–5% of patients with LCL caused by *L. braziliensis* can develop mucosal lesions. Some of these patients did not have treatment during the primary infection, but metastasis can also occur after appropriate treatment [22]. We have followed up with our two pregnant ATL patients for 3–4 years, and they did not relapse or show any signs of mucosal lesions.

## Discussion

Exacerbation of cutaneous lesions in pregnant women due to infection with new- and old-world *Leishmania* spp has been reported; however, the mechanisms accounting for exacerbation of CL have not been clarified [4], [16], [19], [23]. Our results show transient modulation of maternal immune responses during pregnancy as indicated by exacerbated cutaneous lesions, increased parasite burdens, and decreased levels of IFN-γ and NOS2. Of note, both pregnant ATL patients had a restored Th1 response postpartum, associated with healing of the lesions. CL represents the majority of leishmaniasis cases worldwide, and in Brazil, 36% of the infected individuals are women of child-bearing age [22]. Despite the fact that our study was conducted in an endemic area, during an observation period of 4 years, only three pregnant women presented to our clinic with CL, and one of them had an abortion unrelated to leishmaniasis.

The impact of pathogens on human reproduction could be mitigated or exacerbated depending on the time of infection during pregnancy, the maternal immune response, nutritional status, and co-morbidities as described in toxoplasmosis [3]. The number of pregnant women infected with *Leishmania* parasites is generally low, and no information about the frequency of fetal loss in ATL patients is currently available.

At present, it is unclear whether the scarcity of reports on leishmaniasis in pregnancy is due to the use of contraceptives during the antimonial treatment, to increased embryo resorption or fetal abortion, to exclusion of pregnant women in many study designs, or to insufficient data on epidemiology of pregnant women suffering from leishmaniasis, a complex of diseases belonging to the most neglected tropical diseases.

**Key Learning Points**

- Pregnancy is accompanied by transient changes of maternal immunity, exemplified by decreased Th1- and increased Th2-type responses. The changes in innate and adaptive maternal immune responses during pregnancy are tightly linked to gestation and normalize postpartum.
- In patients with leishmaniasis, these transient changes in the maternal immune system enhance susceptibility to infection with *Leishmania* parasites and result in aggravated disease during pregnancy.
- Several drugs used to treat leishmaniasis are teratogenic. But if treatment is necessary, literature points to Amphotericin B as the drug of choice.
- Treatment of visceral leishmaniasis during pregnancy is compulsory, since vertical transmission has been demonstrated.
- As vertical transmission has not been described in cutaneous/mucosal leishmaniasis, patients with ATL may not be treated if the lesions are contained but should be frequently monitored during the course of pregnancy and postpartum.

## Supporting Information

Table S1 Clinical data of ATL patients before specific treatment.
(DOC)

Table S2 Comparison of the in situ inflammatory response in pregnant and nonpregnant ATL patients.
(DOC)
