# Supplementary material for: Two Women Presenting Worsening Cutaneous Ulcers during Pregnancy: Diagnosis, Immune Response, and Follow-up
Source: PLoS Negl Trop Dis. 2013 Dec 12;7(12):e2472. doi: 10.1371/journal.pntd.0002472 (PMC3861112; doi:10.1371/journal.pntd.0002472)
Supplement: Table S1 — Clinical data of ATL patients before specific treatment. (DOC) [file pntd.0002472.s001.doc]

Supporting information Table S1- Clinical data of ATL patients before specific treatment ^a)^

| **Patient** | **Pregnant^b)^** | **Pregnancy**  **stage** | **Age**  **(years)** | **Lesion-size**  **(mm)** | **Lesion-type** | **Lesion site** | **Lesion duration**  **(months)** |
| --- | --- | --- | --- | --- | --- | --- | --- |
| PP1 | yes | 2 months | 24 | 35 x 25 | ulcerated | above knee | 3 |
| PP2 | yes | 2 months | 25 | 22 x 17 | ulcerated | left arm | 2 |
| C1 | no | N/A**^c)^** | 36 | 55X35 | ulcerated | Upper limb | 3 |
| C2 | no | N/A | 26 | ND**^d)^** | ulcerated | trunk | 4 |
| C3 | no | N/A | 24 | 30 x 25 | ulcerated | Upper left forearm | 4 |
| C4 | no | N/A | 26 | 10X8 | ulcerated | Right shoulder | 4 |
| C5 | no | N/A | 24 | 20X15 | ulcerated | Left leg | 6 |
| C6 | no | N/A | 26 | 25X25 | ulcerated | Left forearm | 2 |

**a)** Anti-leishmanial chemotherapy was not prescribed during pregnancy as well as immediately after delivery due to breast-feeding**;** ATL due to infection with *Leishmania braziliensis* was confirmed by parasite detection following standard procedures (VigiLeish – IPEC – Fiocruz).

**b)** Female pregnant and age-matched non-pregnant patients with ATL as well as healthy age-matched female volunteers participated in this study. The pregnant patients were followed up during gestation and partum. **c)-** N/A – not applicable; **d)**-ND – not don
