# Supplementary material for: Two Women Presenting Worsening Cutaneous Ulcers during Pregnancy: Diagnosis, Immune Response, and Follow-up
Source: PLoS Negl Trop Dis. 2013 Dec 12;7(12):e2472. doi: 10.1371/journal.pntd.0002472 (PMC3861112; doi:10.1371/journal.pntd.0002472)
Supplement: Table S2 — Comparison of the in situ inflammatory response in pregnant and nonpregnant ATL patients. (DOC) [file pntd.0002472.s002.doc]

Supporting information Table S2*-* Comparison of the *in situ* inflammatory response in pregnant and non pregnant ATL patients*.

|  | PREGNANT ^a)^  BEFORE  PP1 | AFTER  PP1 | PREGNANT  BEFORE  PP2 | AFTER  PP2 | NONPREGNANT^b)^    Median  (min-max) |
| --- | --- | --- | --- | --- | --- |
| CD3 (%) | 62.6 | 52.9 | 78.1 | 67 | 48(30.2-56.5) |
| CD4 (%) | 44.8 | 36.9 | 36.3 | 34.1 | 34.4 (24.1-39.5) |
| CD8 (%) | 43.5 | 44.5 | 17.4 | 28.8 | 31.1 (9.4-38.8) |
| CD22 (%) | 1.8 | 7.9 | 2 | 27.1 | 5.4 (1.14-13.7) |
| NEU (%) | 7.7 | 12.7 | 4.4 | 0 | 6.5 (1.02-11.5) |
| MØ (%) | 29.9 | 15.9 | 56.3 | 54.1 | 33.2 (10.6-63.8) |
| NOS2 (/++++) | + | ++++ | + | +++ | +++ (++ - ++++) |
| IFN-γ (/++++) | + | ++++ | ++ | +++ | +++ (+ - ++++) |
| IL-10 (/++++) | +++ | +++ | ++ | +++ | ++ (+ - ++++) |
| Foxp3 (%) | 3.5 | 7.8 | 1.23 | 20.8 | 25.9 (6.4-41) |
| Ki-67 (%) | 24.1 | 6.5 | 10.1 | 3 | 9.2 (1-11.4) |
| BCl-2 (%) | 21 | 10.8 | 47.6 | ND | 29.3 (1.2-44.2) |
| BAX (%) | 35 | 58.3 | 56.4 | 52.7 | ND |
| E-selectin | + | + | ++ | + | + (+-+++) |

*Comparison of pregnant ATL patients before and after delivery with a group of

nonpregnant female ATL patients

% - percentage of positive cell in the inflammatory process

/++++- Intensity of positive areas in the tissue. The intensity of NOS2, IFN-γ, IL-10 and E-selectin staining was scored in five microscopic fields (20x· magnification) as discret (+/4 - at least 1 positive area ⁄ field), moderate (++/4 - 2–3 positive areas ⁄ field), intense (+++/4 - 4–5 positive areas ⁄ field) and very intense (++++/4 - >5 positive areas ⁄ field) [11].

1. Biopsies from both pregnant ATL patients were taken at 8 months of pregnancy (BEFORE) and 2-6 months post delivery (AFTER)
2. A group of untreated age- and sex-mated nonpregnant ATL patients with active lesions was used in comparison. The results are expressed as median and as the minimum and maximum count.
